# Indirect choroidal neovascularization secondary to a posterior-segment intraocular foreign body – case report

**DOI:** 10.1186/s12886-020-01437-6

**Published:** 2020-04-21

**Authors:** Yu-Hsiuan Lin, Kai-Ling Peng

**Affiliations:** 1 Department of Ophthalmology, Chi Mei Medical Center, Tainan, Taiwan; 2 Department of Ophthalmology, Kaohsiung Veteran General Hospital, No.386, Dazhong 1st Rd., Zuoying Dist., Kaohsiung City, 81362 Taiwan, Republic of China

**Keywords:** Intraocular foreign body (IOFB), Choroidal neovascularization (CNV), Intravitreal injection (IVI)

## Abstract

**Background:**

We reported a rare case of indirect choroidal neovascularization (CNV) secondary to a posterior-segment intraocular foreign body (IOFB) that was not located in the area of direct injury but in the fovea. After intravitreal injections (IVIs) of aflibercept, the choroidal neovascularization (CNV) lesion disappeared and vision improved.

**Case presentation:**

A 26-year-old male patient suffered from a fast-shot metallic IOFB in his right eye. He underwent primary corneal repair, pars plana vitrectomy, IOFB removal and an IVI of antibiotics in the right eye. Two weeks later, cataract surgery was performed on the right eye for traumatic cataract after an episode of acute phacolytic glaucoma. The best-corrected visual acuity (BCVA) of the right eye improved to 20/20 5 months after the first surgery. However, the vision of the right eye worsened suddenly with metamorphopsia 1 year after the first surgery. Color fundus images showed a whitish lesion with faint retinal hemorrhage and surrounding sensory elevation. Fluorescein angiography (FA) revealed a lesion with early- and late-phase severe leakage. Optical coherence tomography (OCT) demonstrated a CNV lesion with surrounding subretinal fluid. The patient received an IVI of aflibercept every 8 weeks for 3 times. Finally, the BCVA of the right eye improved to 20/25.

**Conclusions:**

For rare cases of fovea-spared injury by a metallic IOFB, it is still necessary to pay close attention to the foveal microstructure to avoid possible CNV formation. Treatment with IVIs of anti-VEGF, aflibercept, as early as possible could provide good visual outcomes.

## Background

Choroidal neovascularization (CNV) secondary to traumatic choroidal rupture after direct penetrating injuries has been reported to manifest between 1 month and 4 years after ocular trauma [1]. Generally, the retina, choroid, and Bruch’s membrane are lacerated at the time of impact by a fast-shot intraocular foreign body (IOFB). Injury to Bruch’s membrane from an IOFB results in a defect where CNV is derived from the choriocapillaries and grows into the subretinal or subpigment epithelial space.

For traumatic CNV, anti-vascular endothelial growth factor (anti-VEGF) agents are effective as they bind to the VEGF induced by CNV lesions, thereby leading to a direct angiostatic effect to further resolve surrounding edema. Anti-VEGF agents include bevacizumab, an early anti-VEGF agent that is a full IgG1 antibody; ranibizumab, which is a monoclonal humanized antibody fragment; and aflibercept, a more recent anti-VEGF agent that is a VEGF receptor 1/2 Fc fusion protein.

We reported a rare case of indirect CNV secondary to a posterior-segment IOFB that was not located at the area of direct injury but in the fovea. After 3 IVIs of aflibercept, the CNV lesion disappeared and vision improved.

## Case presentation

**Fig. 1:**
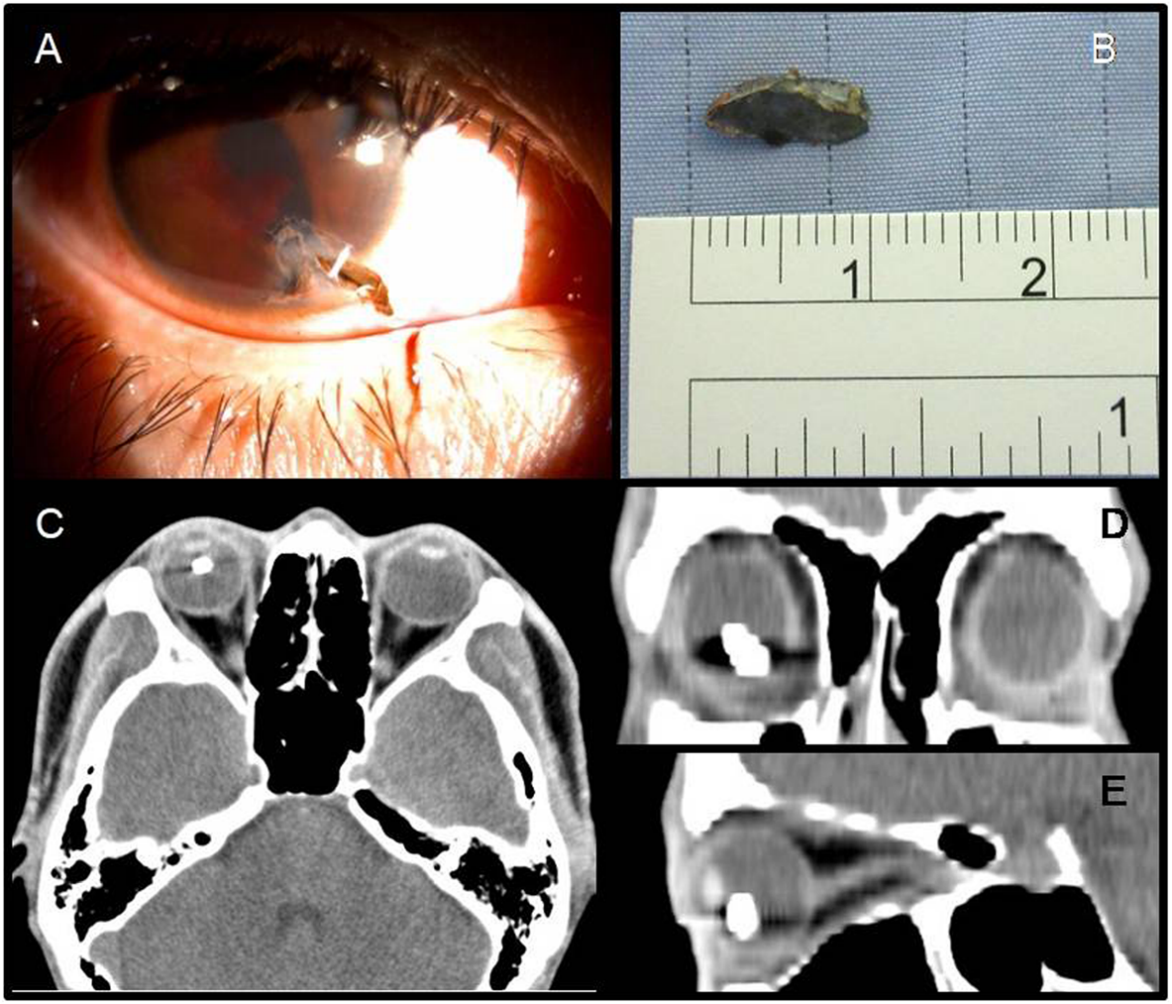
Under a slit lamp, the patient was found to have a diffuse, thin hyphema and a blood clot at the position of the distorted and torn pupil (**a**). The metallic IOFB was approximately 8X4 mm in size (**b**). Orbital computed tomography (CT) without contrast enhancement showed the metallic IOFB floating in the vitreous cavity in different views, such as the horizontal (**c**), coronary (**d**) and sagittal (**e**) views

A 26-year-old male patient without a history of systemic diseases or myopia suffered from a fast-shot metallic IOFB in his right eye while working. He was sent to our emergency room, where a penetration wound with iris incarceration was located at the nasal lower cornea in his right eye. Under a slit lamp, he was found to have a diffuse, thin hyphema and some blood clots at the position of the distorted and torn pupil (Fig. 1a). His best-corrected visual acuity (BCVA) was hand motion at 30 cm in the right eye and 18/20 in the left eye. Orbital computed tomography (CT) without contrast enhancement showed a metallic IOFB with a size of 8X4 mm (Fig. 1b) floating in the vitreous cavity in different views, such as the horizontal (Fig. 1c), coronary (Fig. 1d) and sagittal views (Fig. 1e).

He underwent primary corneal repair, pars plana vitrectomy, IOFB removal and prophylactic IVI of antibiotics with vancomycin (1 mg/0.1 c.c.) and ceftazidime (2 mg/0.1 c.c.) at the end of surgery. At the beginning of vitrectomy, no retinal impact was noted. However, the IOFB dropped to the nasal and upper retinal area outside the macula while performing the vitrectomy to release vitreous traction around the IOFB. Two weeks later, his intraocular pressure in the right eye elevated abruptly to 30 mmHg with cell 3+ in the deep anterior chamber and a cloudy, swollen lens. Because of the suspicion of phacolytic glaucoma, he underwent cataract surgery. The BCVA in his right eye recovered to 20/20 5 months later, with good foveal contour except for a small retinal dimple (yellow arrow) and a segment of hyporeflectivity at the inner segment and outer segment (IS/OS) junction (white arrow) of the temporal fovea (Fig. 3a).

**Fig. 2:**
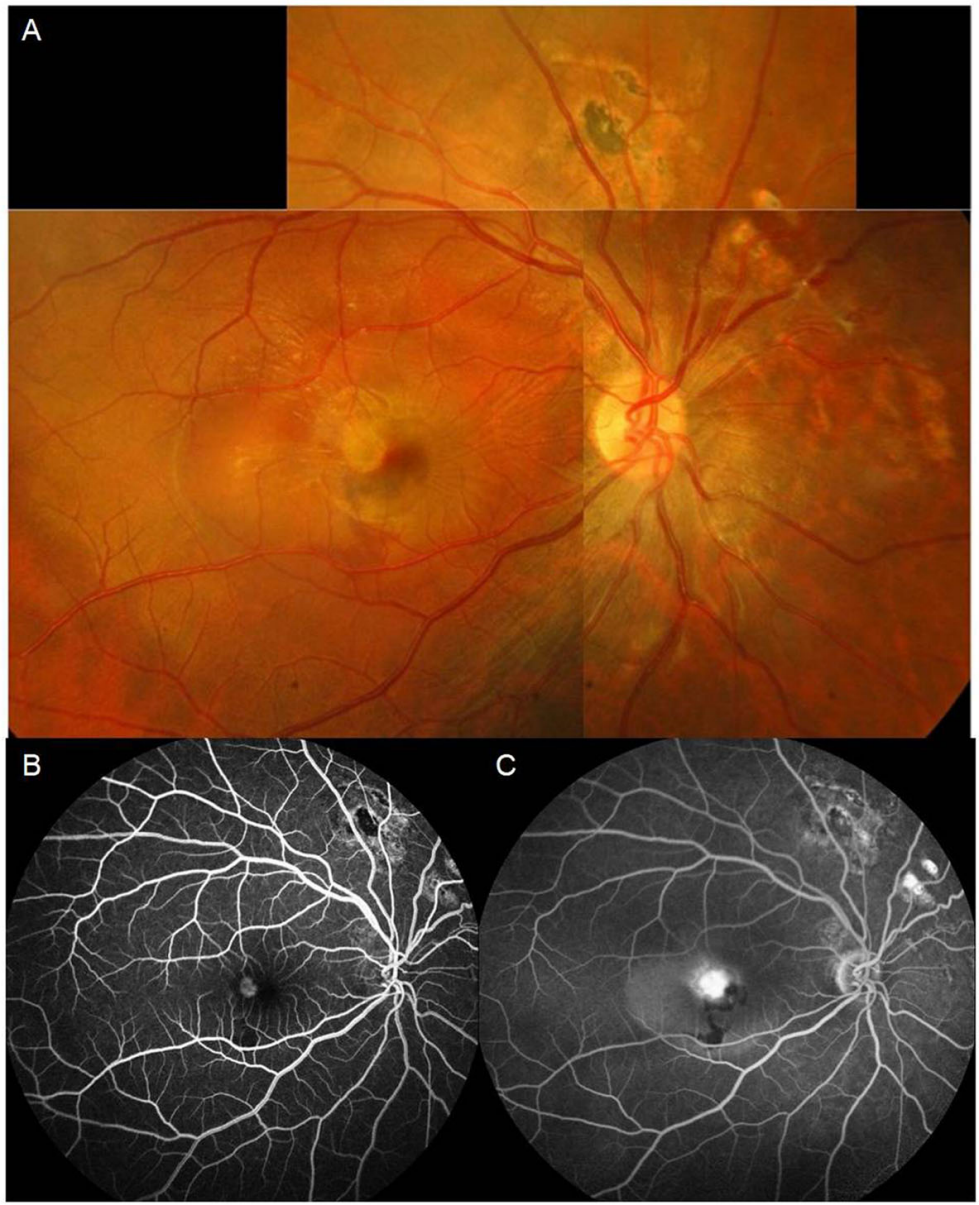
There was a whitish lesion at the fovea with faint retinal hemorrhage and surrounding sensory elevation (**a**). FA demonstrated that the foveal CNV was hyperfluorescent and leaking at the early phase (**b**) with severe dye pooling at the late phase (**c**)

**Fig. 3:**
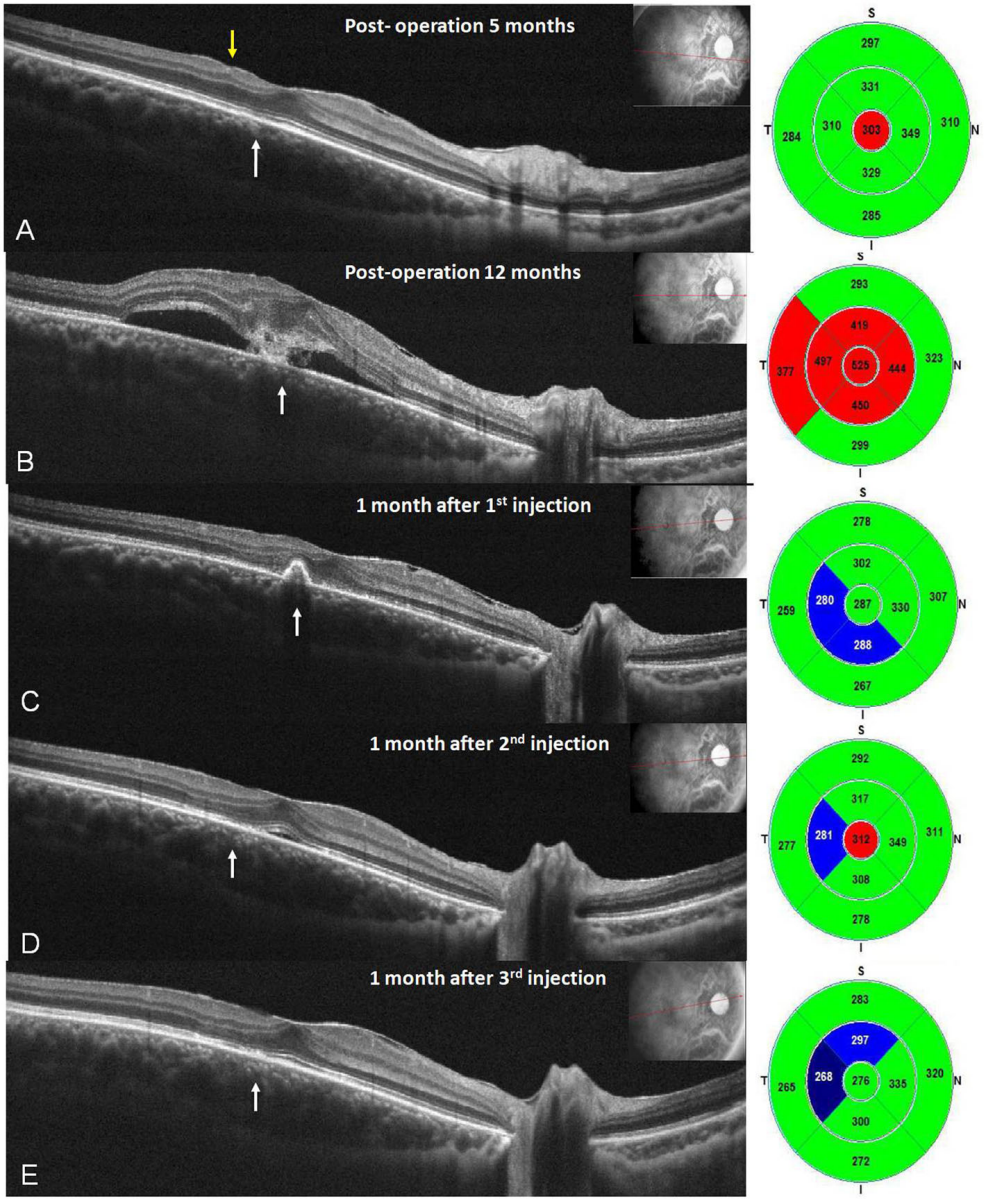
Five months after the first surgery, OCT revealed good foveal contour with a small retinal dimple (yellow arrow) and a small defect of the outer segment of the photoreceptors (white arrow) in the temporal parafoveal area (**a**). Twelve months after the first surgery, he complained of a sudden onset of blurred vision with metamorphopsia, with OCT showing subfoveal fluid and a CNV lesion (**b**). After he received the first intravitreal injection of aflibercept (4 mg/0.1 mL) in his right eye, OCT showed total resolution of subretinal fluid with residual CNV lesions 8 weeks later (**c**). After the second injection, OCT demonstrated an even, flat retinal pigmented epithelium layer with residual subfoveal fluid 8 weeks later (**d**). After the third injection, OCT presented no more subfoveal fluid, with vision improvement to 16/20 8 weeks later (**e**)

Twelve months after the first surgery, he returned to our clinic because of a sudden onset of metamorphopsia with a decline in BCVA to 6/20 in his right eye. There was a whitish lesion at the fovea with faint retinal hemorrhage and surrounding sensory elevation (Fig. 2a). Optical coherence tomography (OCT) revealed subretinal fluid with a CNV lesion (Fig. 3b). Fluorescence angiography (FA) demonstrated that the foveal CNV was hyperfluorescent and leaking at the early phase (Fig. 2b) with severe dye pooling at the late phase (Fig. 2c). He received an intravitreal injection of aflibercept (4 mg/0.1 mL) in his right eye every 8 weeks for three times; over the treatment period, OCT showed a gradual reduction of the subretinal fluid and the resolution of the CNV lesion, which completely disappeared (Fig. 3c, d, e). His right-eye BCVA improved to 16/20 without visual distortion within 3 months.

## Discussion and conclusions

Choroidal rupture, which may later result in CNV, could be present in posterior-segment injuries induced from open-globe trauma or even closed-globe trauma [2]. Regarding choroidal rupture induced by penetrating trauma, direct injury damages the original structures of the wall of the eyeball. The mechanism of blunt trauma-related choroidal rupture includes the deformation of the globe during high-speed impact and shearing force along the eyeball tissue [3]. Approximately 10–20% of patients may develop CNV months to years after [1]. In addition, indirect choroidal rupture may be related to chronic inflammation and the consequent abnormal production of proangiogenic mediators [4].

In our case, the IOFB was in the middle of the vitreous cavity as shown in three different views of orbital CT. During surgeries, there was no retinal damage over the posterior pole except in the nasal and upper retinal areas, which was caused by the IOFB dropping and rolling during its removal. In the OCT before CNV formation, there was a small retinal dimple and a segment of hyporeflectivity at the photoreceptor inner segment and outer segment (IS/OS) junction of the temporal fovea where the CNV formed later. We assumed that the breakage of the photoreceptor layer was caused by an inflammatory reaction, the toxicity of the metallic molecules from the IOFB, or the small mechanical shearing force as the accident occurred.

In cases of ocular siderosis and subretinal hemorrhage, ferrous iron generates radicals and causes oxidative stress to the photoreceptors or the RPE [5]. Long-term retention of iron-containing foreign bodies has even been reported to cause retinal pigment epithelial defects [6]. In our case, the metallic IOFB stayed in the eye less than 24 h, and the residual concentration of metallic molecules was too low to cause severe harm, although microinjury to the retina or a small break at the level of the IS/OS junction could not be excluded.

Referring to the treatment of traumatic CNV, Chen [7] et al. administered IVIs of bevacizumab for three consecutive months in a patient with direct foveal CNV due to a posterior-segment IOFB without recurrence. Fernández-López [8] et al. performed IVIs of bevacizumab for four consecutive months in a patient with direct foveal CNV whose injury was caused by a posterior-segment IOFB with recurrence, and additional injections were administered later. Liang F [9]. treated a patient with direct foveal CNV after contusion-induced Bruch’ membrane rupture with a one-time IVI of ranibizumab without recurrence. According to the previous studies about intraocular VEGF factor A (VEGF-A) suppression times (VSTs) under treatment of aflibercept for neovascular age-related macular degeneration (nAMD), the mean VST was considerably longer averaging 71 days [10]. In our patient, for indirect foveal CNV following a posterior-segment IOFB, we administered an IVI of aflibercept every 8 weeks for 3 times without recurrence. Further investigations are needed to establish the best dosing regimen in these patients with traumatic CNV.

CNV may occur later in cases of obvious foveal choroidal rupture or Bruch’s membrane rupture after posterior-segment IOFB. For the rare cases of fovea-spared injury by a metallic IOFB, it is still necessary to pay close attention to the foveal microstructure at each follow-up visit to avoid possible CNV formation. Regardless of whether CNV is direct or indirect, prompt treatment with an IVI of an anti-VEGF, such as aflibercept, as early as possible could truly provide good visual outcomes.

## Data Availability

All data supporting our findings will be shared upon request, although the majority is contained within the manuscript.

## Abbreviations

CNV: Choroidal Neovascularization
IOFB: Intraocular Foreign Body
BCVA: Best-corrected visual acuity
FA: Fluorescein angiography
OCT: Optical coherence tomography
IVI: Intravitreal injection
anti-VEGF: Anti-vascular endothelial growth factor
VEGF-A: Anti-vascular endothelial growth factor A
VSTs: Anti-vascular endothelial growth factor A suppression times
nAMD: Neovascular age-related macular degeneration
