# Correction to: Indirect choroidal neovascularization secondary to a posterior-segment intraocular foreign body – case report

**DOI:** 10.1186/s12886-020-01450-9

**Published:** 2020-05-04

**Authors:** Yu-Shiuan Lin, Kai-Ling Peng

**Affiliations:** 1 Department of Ophthalmology, Chi Mei Medical Center, Tainan, Taiwan; 2 Department of Ophthalmology, Kaohsiung Veteran General Hospital, No.386, Dazhong 1st Rd., Zuoying Dist, Kaohsiung City, 81362 Taiwan, Republic of China

**Correction to: BMC Ophthalmol 20, 161 (2020)**

**https://doi.org/10.1186/s12886-020-01437-6**

After publication of our article [1] we were notified that one of the author names was misspelled.

Incorrect author name:

- Yu-Hsiuan Lin

Correct author name:

- Yu-Shiuan Lin

The original article has been corrected.
